# Patterns of association of native and exotic boring polychaetes on the southeastern Pacific coast of Chile: the combined importance of negative, positive and random interactions

**DOI:** 10.7717/peerj.8560

**Published:** 2020-04-24

**Authors:** Paula E. Neill, Nicolás Rozbaczylo, Cristóbal Villaseñor-Parada, Garen Guzmán-Rendón, Sandra Sampértegui, Cristián E. Hernández

**Affiliations:** 1 Laboratorio de Ecología Evolutiva y Filoinformática, Departamento de Zoología, Facultad de Ciencias Naturales y Oceanográficas, Universidad de Concepción, Concepción, Chile; 2 Colegio Almondale Lomas, Lomas de San Sebastián, Concepción, Chile; 3 Faunamar Ltda, Consultorías Medio Ambientales e Investigación Marina, Santiago, Chile; 4 Laboratorio de Invasiones Biológicas (LIB), Facultad de Ciencias Forestales, Universidad de Concepción, Concepción, Chile; 5 Universidad Católica de Santa María, Arequipa, Perú

**Keywords:** Biological invasions, Biodiversity, Biotic resistance, Invasion ecology, Non-indigenous species, Polydora, Southeastern Pacific coast

## Abstract

**Background:**

Studies of biological invasions focus on negative interactions between exotic and native biotas, emphasizing niche overlap between species and competitive exclusion. However, the effects of positive interactions and coexistence are poorly known. In this study we evaluate the importance of positive, negative, or random species associations in explaining the coexistence of native and exotic boring polychaetes inhabiting invertebrate hosts, on the southeastern Pacific coast of Chile. We assess three hypotheses to explain the observed patterns: positive species interactions, weak competitive interactions, and competitive intransitivity.

**Methodology:**

To assess the potential effect of competition between native and exotic polychaetes we analyzed patterns of co-occurrence of species pairs in northern and southern regions, using the metric of the probabilistic model. Since biotic interactions can affect not only native species, we also evaluated correlations between native and exotic polychaete abundance, using reduced major axis regression linear models. To assess the transitivity of competitive hierarchies we used metrics and analytical methods based on abundance matrices to estimate species competition and patch transition matrices.

**Results:**

On average 50% of the species pairs presented significant weak negative associations, all associated with the exotic species *Polydora rickettsi*; the remaining 50% had random associations, and none showed positive associations. However, the relationship of abundance between native and exotic boring polychates supports a tendency towards coexistence. At local and regional scales, we observed the presence of a transitive network competition structure, where the exotic boring polychaete, *P. rickettsi* was generally the dominant species.

**Conclusions:**

Our results support that native and exotic boring polychaete species coexist through weak competitive interactions. Nevertheless, the large number of random interactions observed indicates that species coexistence can be accounted for by stochastic processes, as proposed by neutral theory. Coexistence may be a frequent result of interactions between native and exotic species, although less apparent than competitive exclusion. Thus, the probabilistic point-of-view used here provides a statistical tool for evaluating coexistence as a result of exotic and native species’ interactions, an idea which has been proposed in invasion ecology, but largely lacks empirical support and methodologies for detecting underlying mechanisms. Finally, we found evidence that *P. rickettsi* is a successful invader by being competitively dominant, but not excluding other species.

## Introduction

Studies on biological invasions have mainly focused on the importance of negative interactions between non-indigenous species and native biota (e.g., Elton, 1958; Seabloom et al., 2003; Olyarnik et al., 2009). According to the biotic resistance hypothesis (e.g., Catford, Jansson & Nilsson, 2009), native biota can deter exotic species invasion, through competition and predation as local mechanisms (Elton, 1958; Shea & Chesson, 2002; Byers & Noonburg, 2003; Olyarnik et al., 2009). However, biotic resistance is frequently evaluated as the relationship between native and exotic species diversities (i.e., diversity resistance hypothesis), where a negative relationship is interpreted as biotic resistance of the native community to invasion success (Elton, 1958; Kennedy et al., 2002; Herben et al., 2004; Fargione & Tilman, 2005; Fridley et al., 2007). Nevertheless, apparent contradictions in empirical studies have led researchers to discuss the importance of other interactions, such as the influence of facilitation on population- and community-level variables (Bruno, Stachowicz & Bertness, 2003).

Negative relationships may be observed at the local scale, due to negative biotic interactions, while at a regional scale the overall relationship may be positive, since regional heterogeneity in abiotic conditions may present more niche opportunities for both native and exotic species richness (Shea & Chesson, 2002; Byers & Noonburg, 2003). Particularly in cases where negative interactions do not totally exclude exotics, biotic resistance may act to constrain the abundance of established exotics (Levine, Adler & Yelenik, 2004; Olyarnik et al., 2009). On the other hand, biotic resistance can also be explained by differentiation in the occupation of niches or fitness between native and invasive species (Byun, De Blois & Brisson, 2017). This is inferred from the classical theory of competitive interactions, where limits in niche overlap, or the use of resources between native species and invasive species, determine the results of the interspecific interaction (MacArthur & Levins, 1967; Weltzin et al., 2003). According to this theory, an invading species will not establish where a resident species occupies a similar niche or has similar traits. Therefore, when there is niche overlap, the fitness difference between the resident species and the invader determines which one will be competitively excluded (MacDougall, Gilbert & Levine, 2009). This idea implicitly assumes that all resident species have negative effects on invader success, and that only the negative interactions and abiotic factors can deplete populations and remove species, which largely ignores the effect of other ecological interactions, or the possibility of no (or incomplete) exclusion. In fact, it has been suggested that the coexistence of native and invasive species depends on the number and strength of simultaneous negative interactions (Mills, Rader & Belk, 2004). Moreover, the competitive exclusion assumption is unlikely to be true in natural communities given that species that repel invaders are as common as the species that facilitate their colonization (Henriksson et al., 2016), and exclusion could occur if only one limiting resource exists or if a single species is a superior competitor for several resources (e.g., Seabloom et al., 2003). Consequently, the coexistence of native and invasive species could be a frequent result in natural communities; changing the general question of how do native species exclude exotics (biotic resistance hypothesis), for the complementary question of how do native and exotic species coexist? The first intuitive answer to the latter question is: a predominance of positive species interactions (i.e., facilitation) can allow native and exotic species to coexist (Bruno, Stachowicz & Bertness, 2003). However, there is a second answer that is in agreement with both the competitive exclusion principle (Gause, 1934) and the biotic resistance hypothesis, which proposes that these species coexist due to weak competitive interactions (e.g., Liao, Bogaert & Nijs, 2015). Furthermore, a third hypothesis suggests that competitive intransitivity networks (i.e., non-linear hierarchical competition, in which there is no single best competitor) allow for species co-existence (Soliveres et al., 2015; Ulrich, Jabot & Gotelli, 2017), even under modest levels of intransitivity (Laird & Schamp, 2006).

Species are either positively, negatively, or randomly associated with one another, according to their niches (Sfenthourakis, Tzanatos & Giokas, 2006; Gotelli & Ulrich, 2010; Veech, 2013). Therefore, coexistence can result from three potential outcomes of native and exotic species’ interactions: (1) facilitation through positive species associations (Hypothesis 1); (2) weak competitive or neutral interactions through weak negative species associations with hierarchical competition (Hypothesis 2); and (3) if competitive intransitivity is promoting native and exotic species coexistence the ecological guild will show significantly strong negative species associations, with non-hierarchical competition (i.e., non transitivity or competitive networks, where species are ambiguous in their order of competitive abilities) (Hypothesis 3). We evaluated each of these hypotheses using as a study model the guild of native and exotic boring polychaetes present in calcareous hosts on the southeastern Pacific coast of Chile.

Evaluating these hypotheses is especially important for the southeastern Pacific coast, where competitive exclusion (i.e., the biotic resistance hypothesis) has been proposed as one of the principal explanations for low invader success (Castilla & Neill, 2009). Preliminary analysis of invasion patterns on the southeastern Pacific coast suggests that the number and impact of exotic species is lower than on other shores in both the northern and southern hemispheres (Castilla & Neill, 2009). Nevertheless, deliberate introduction of species for aquaculture is on the rise (e.g., Camus, 2005; Gozlan, 2017), and currently there are land-based and water-based aquaculture facilities in both northern and southern Chile. This is worrisome for the southeastern Pacific coast considering that, globally, aquaculture activities are considered an important gateway and reservoir for exotic species (Naylor, Williams & Strong, 2001). Boring polychaetes are common epibionts on these mollusks, as well as on other invertebrate species with calcareous shells, both in culture facilities and natural environments (Moreno, Neill & Rozbaczylo, 2006; Diez, Radashevsky & Orensanz, 2011). A review by Moreno, Neill & Rozbaczylo (2006) reports nine species of boring polychaetes present in calcareous substrata along the coast of Chile, of which at least six species were non-indigenous. These researchers report that the relationships between boring polychaete species and their hosts are not highly specific, in accordance with the general pattern for shell-boring polychaetes (Simon & Sato-Okoshi, 2015), with several examples of native and exotic boring polychaete species coexisting and utilizing multiple hosts. Although boring polychaetes utilize the same type of habitat, potential ecological interactions between exotic and native polychaetes are unknown and represent an interesting study model to evaluate whether the apparently low success of invasive species on the southeastern Pacific coast is due to negative interactions. The aim of this study is to evaluate the importance of positive, negative, or random species associations to explain the coexistence of native and exotic boring polychaetes inhabiting invertebrate hosts on the southeastern Pacific coast of Chile.

## Materials & Methods

### Boring polychaete sampling, processing, and quantification

**Figure 1:**
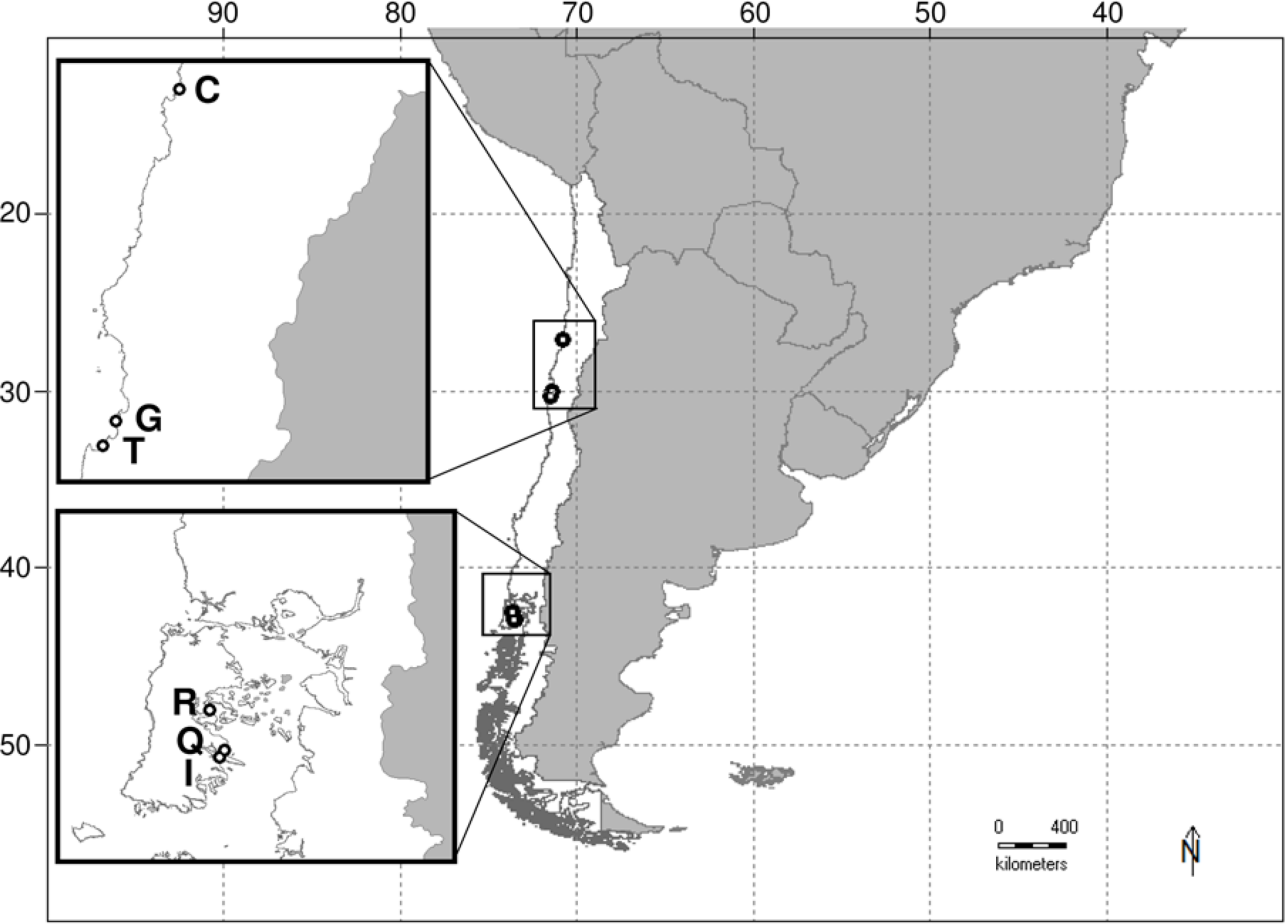
Map of study area on the southeastern Pacific coast of Chile. Letters adjacent to unfilled circles on map insets indicate the study sites from northern (upper) and southern (lower) regions: C = Caldera (−27°2′26.4″, −70°49′4.9″); G = Guanaqueros (−30°1′55.5″, −71°23′23.5″); T = Tongoy (−30°15′27.9″, −71°30′24.3″); R = Rilan (−42°32′9.2″, −73°37′53.4″); Q = Queilen (−42°53′13.3″, −73°30′17.7″); and I = Isla Tranqui (−42°56′49.3″, −73°32′52.1″).

Boring polychaetes were obtained from mollusk hosts present in and near aquaculture centers at three sites in the northern region and three sites in the southern region along southeastern coast of Chile (Fig. 1). Native host shells were collected by hand while hookah diving (i.e., a diving system where air is supplied from a hose attached to an air compressor located on land or on a boat) or SCUBA diving, and transported to the laboratory where they were preserved in 10% formalin for subsequent boring polychaete extraction. Exotic host shells (*Crassostrea gigas* Thunberg, 1793) were obtained from aquaculture centers present at each of the sites, and a total of 784 shells of 19 species were collected and processed. To extract the boring polychaetes each individual shell was chemically treated, with initial submersion in 5% nitric acid (HNO_3_) for a period of 12 h, and later neutralized with 5% anhydrous sodium sulfate (NH_2_SO_4_) for an additional 12 h. This process dissolves the calcite crystals of the shell without damage to the boring polychaetes, which were then separated by hand under a dissecting microscope for identification and quantification. Species identification was made by drawing diagnostic structures using a camera lucida installed on a stereoscopic microscope (Leica model M3Z) and using keys by Gravier (1908), Fauchald (1977), Rozbaczylo (1980), Rozbaczylo (1985) and Fitzhugh & Rouse (1999). Identification was facilitated by bibliographic information on native and exotic boring polychaetes from different sources, especially for Spionidae polychaetes (Rozbaczylo, Schmiede & Sánchez, 1980; Rozbaczylo, Méndez & Bravo, 1994; Basilio, Cañete & Rozbaczylo, 1995; Sato-Okoshi & Takatsuka, 2001; Cárdenas & Cañete, 2004; Radashevsky & Olivares, 2005; Bertrán, Vargas & Quijón, 2005; Moreno, Neill & Rozbaczylo, 2006) and Cirratulidae polychaetes of the genus *Dodecaceria* (Carrasco, 1977; Rozbaczylo & Carrasco, 1996; Moreno, Neill & Rozbaczylo, 2006). Boring polychaete species were classified as native or exotic according to the identification and records published by Orensanz et al. (2002), Castilla et al. (2005), Moreno, Neill & Rozbaczylo (2006) and Çinar (2012).

### Analyses of boring polychaete co-occurrence and abundance

Analyses were conducted using only infected hosts (i.e., mollusk shells with boring polychaete abundance >0) as suggested by Rózsa, Reiczigel & Majoros (2000). Details of the number of exotic and native boring polychaetes in hosts sampled from the southern Pacific coast of Chile are contained in the Supplemental Data Base S1. To evaluate the importance of positive, negative, or random species associations in the coexistence or potential exclusion of species, we used a co-occurrence analysis based on the probabilistic model proposed by Veech (2013) (see also Griffith, Veech & Marsh, 2016). In this model one can obtain the probability of co-occurrence between species pairs under the condition where a species’ probability of occurrence at each site is equal to its observed frequency among all the sites, allowing one to analytically obtain the probability (*P*) that two selected species co-occur (i.e., co-occurrence that is significantly large and greater than expected is a positive association (Hypothesis 1); co-occurrence that is significantly small and less than expected is a negative association; or co-occurrence that is not significantly different from expected is a random association).

We calculated the co-occurrence among boring polychaetes in individual hosts from two different regions (northern and southern regions). Each presence/absence matrix of polychaetes in individual hosts was analyzed using the co-occur package (Griffith, Veech & Marsh, 2016) implemented in R Code (See Supplemental RScript S2). This function uses community data (e.g., species by site matrix or vice-versa) to produce: (a) the probabilities (*p*-values) that a given species pair co-occurs less than (p lt) or greater than (p lg) expected by chance; (b) evaluation of the contribution of individual species to observed patterns, based on the *p*-values and a species association pairing profile; and (c) quantification of the strength of positive and negative associations between species pairs to determine if there is evidence of biotic resistance between native and exotic polychaetes as defined by Griffith, Veech & Marsh (2016), the latter analysis measures the differences between expected and observed frequency of co-occurrence standardized by dividing the number of sample sites in the dataset, where positive values indicate positive associations and negative values indicate negative ones (values are bounded from −1 to 1).

Since co-occurrence analyses only consider the presence or absence of a species, which does not allow for the inference of biotic interactions mediated by organism abundances, we also evaluate the general effect of non-complete species exclusion using a reduced major axis regression (RMA) linear models to evaluate the relationship between native and exotic polychaete abundance, considering error in both variables, and no assumption of a dependent–independent variable relationship. The significance of the relationship was evaluated using PAST software version 3.25 (Hammer, Harper & Ryan, 2001). In this analysis a significant positive slope indicates a coexistence trend, a negative slope indicates an exclusion trend, and a non-significant slope indicates a random association. Second, to assess the transitivity of hierarchical competition (Hypothesis 2) (Hypothesis 3) we used analytical methods based on abundance matrices to estimate species competition and patch transition matrices following the approach by Ulrich et al. (2014), implemented in *Transitivity* software (Ulrich, 2012). This approach is based on a randomization test to evaluate the degree of intransitivity from patch transition matrices (P) in combination with empirical or simulated competition C matrices. Thus, a single metric of intransitivity was calculated for each host, and at equilibrium observed species abundances should be equal to the dominant eigenvector of a hypothetical species x species transition matrix (i.e., the matrix that contains the probability that one species replaces another in a given host). We randomly generated 500,000 patch-transition species by species matrices, of which the 500 best fitting ones (i.e., matrices where the dominant eigenvector is closest to the observed species abundances) were chosen for the calculation of confidence limits and metrics. To quantify transitivity in the transition matrix P, we use the *τ*_p_ metric, which runs from 0.0 (completely intransitive) to 1.0 (fully transitive). To evaluate the importance of the different hypotheses at different spatial scales, all analyses were conducted at a regional level (i.e., combining northern and southern sites), as well as separately for different regions.

## Results

### Boring polychaetes

We found a total of four boring polychaete species inhabiting 13 different host species with calcareous shells, with a total of 254 infected host individuals (32% prevalence). We identified two native boring polychaete species: *Dipolydora huelma* (Sato-Okoshi & Takatsuka, 2001) and *Dodecaceria opulens* Gravier, 1908 and two exotic boring polychaete species: *Boccardia tricuspa* (Hartman, 1939) and *Polydora rickettsi* Woodwick, 1961.

### Co-occurrence

**Figure 2:**
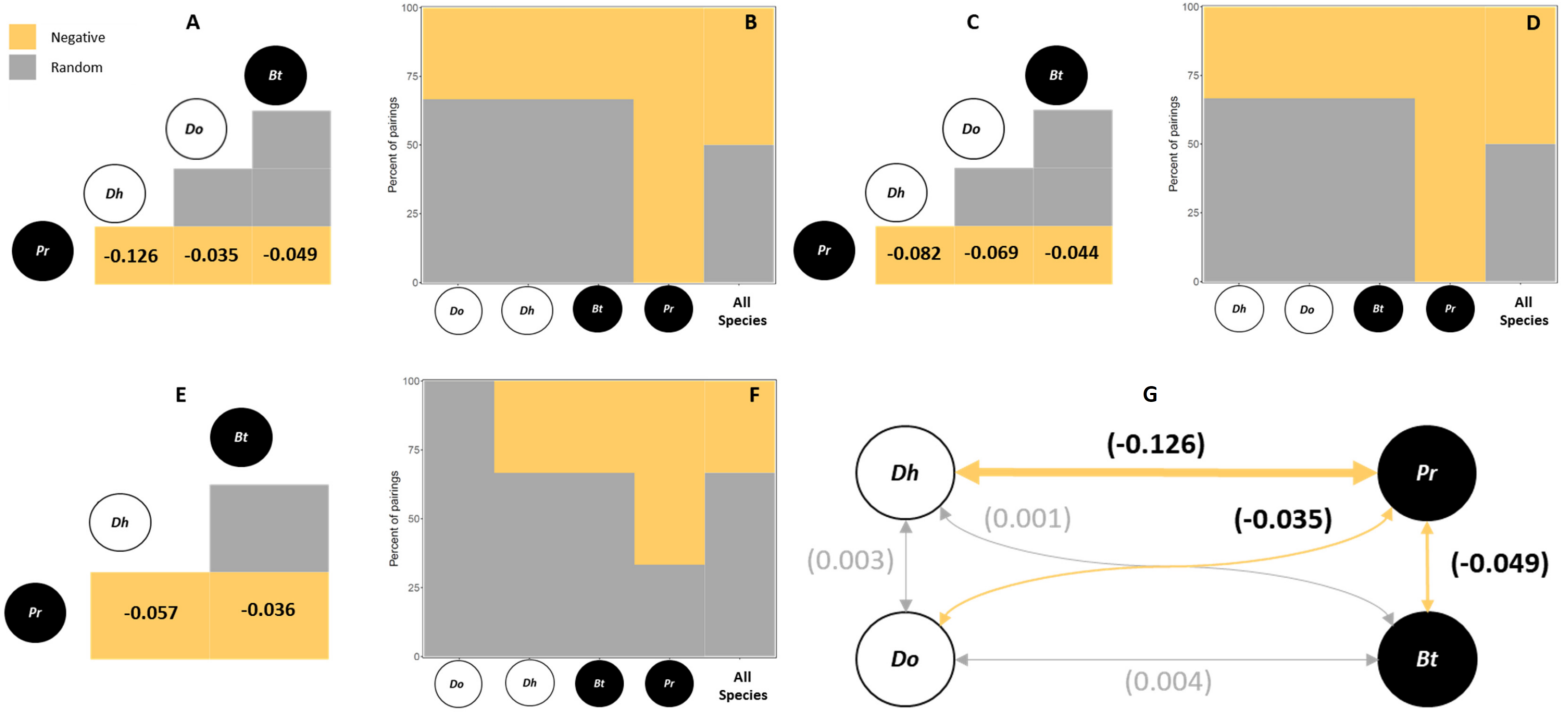
Probabilistic Species Co-Occurrence Analysis performed. For all hosts and sites, including (A) the species co-occurrence matrix, indicating the significative negative co-occurrence patterns and the strength of significative associations between species pairs (values are bounded from −1 to 1, yellow squares with negative values indicate negative associations, grey squares indicate random associations), and (B) the species association profile, indicating the contribution of individual species to observed patterns; all hosts from the Southern area, including (C) the species co-occurrence matrix, and (D) the species association profile; and all hosts from the Northern area, including (E) the species co-occurrence matrix, and (F) the species association profile. The diagram (G) shows the general strength of negative associations between species pairs, where the two native boring polychaete species are *Dipolydora huelma* (indicated as ***Dh***) and *Dodecaceria opulens* (indicated as ***Do***), and the two exotic boring polychaete species are *Boccardia tricuspa* (indicated as ***Bt***) and *Polydora rickettsi* (indicated as ***Pr***).

For all hosts species and sampling sites, 50% of the species pairs had negative associations, (in northern sites only 33% of species pairs had negative associations) (i.e., *p* < 0.05). The remaining 50% of species pairs (67% in the north) had random associations (Table 1, Fig. 2). No positive associations were observed in these analyses. The main species that contributed to the observed patterns was the exotic polychaete *P. rickettsi*, which showed negative co-occurrence with all other polychaete species (Fig. 2). The quantification of the strength of negative associations between *P. rickettsi* and the other species showed weak competitive interactions where the most affected species was the native *D. huelma,* followed by *D. opulens* and *B. tricuspa* (Fig. 2A).

**Table 1:** Summary of probabilistic Species Co-Occurrence Analysis of native and exotic boring polychaetes in individual hosts. Performed for: (A) all hosts and sites, (B) all hosts from Southern sites, and (C) all hosts from Northern sites. Bold numbers indicate significant negative (p lt) co-occurrence patterns.

A) ALL HOSTS AND SITES								
sp1	sp2	sp1 inc	sp2 inc	obs cooccur	prob cooccur	exp cooccur	p lt	p gt
*Dipolydora_huelma*	*Dodecaceria_opulens*	89	40	12	0.05	13.3	0.38609	0.74478
*Dipolydora_huelma*	*Boccardia_tricuspa*	89	34	14	0.042	11.3	0.89005	0.19815
*Dipolydora_huelma*	*Polydora_rickettsi*	89	182	27	0.227	60.7	**<0.00001**	1.00000
*Dodecaceria_opulens*	*Boccardia_tricuspa*	40	34	6	0.019	5.1	0.77186	0.40052
*Dodecaceria_opulens*	*Polydora_rickettsi*	40	182	18	0.102	27.3	**0.00086**	0.99975
*Boccardia_tricuspa*	*Polydora_rickettsi*	34	182	10	0.087	23.2	**<0.00001**	1.00000
**B) SOUTHERN SITES**								
**sp1**	**sp2**	**sp1 inc**	**sp2 inc**	**obs cooccur**	**prob cooccur**	**exp cooccur**	**p lt**	**p gt**
*Dipolydora_huelma*	*Dodecaceria_opulens*	69	22	12	0.125	13.8	0.25868	0.87104
*Dipolydora_huelma*	*Boccardia_tricuspa*	69	20	10	0.114	12.5	0.14801	0.93875
*Dipolydora_huelma*	*Polydora_rickettsi*	69	43	18	0.245	27	**0.00031**	0.99994
*Dodecaceria_opulens*	*Boccardia_tricuspa*	22	20	6	0.036	4	0.93406	0.17516
*Dodecaceria_opulens*	*Polydora_rickettsi*	22	43	1	0.078	8.6	**0.00008**	1.00000
*Boccardia_tricuspa*	*Polydora_rickettsi*	20	43	3	0.071	7.8	**0.01164**	0.99783
**C) NORTHERN SITES**								
**sp1**	**sp2**	**sp1 inc**	**sp2 inc**	**obs cooccur**	**prob cooccur**	**exp cooccur**	**p lt**	**p gt**
*Dipolydora_huelma*	*Dodecaceria_opulens*	20	18	0	0.015	2.3	0.07094	1.00000
*Dipolydora_huelma*	*Boccardia_tricuspa*	20	14	4	0.012	1.8	0.97988	0.08604
*Dipolydora_huelma*	*Polydora_rickettsi*	20	139	9	0.116	17.9	**<0.00001**	1.00000
*Dodecaceria_opulens*	*Boccardia_tricuspa*	18	14	0	0.01	1.6	0.16357	1.00000
*Dodecaceria_opulens*	*Polydora_rickettsi*	18	139	17	0.104	16.1	0.87565	0.41789
*Boccardia_tricuspa*	*Polydora_rickettsi*	14	139	7	0.081	12.6	**0.00007**	1.00000

**Notes.**

Sp1 inc Number of sites (or samples) that have species 1 sp2 inc number of sites that have species 2 obs co-occur observed number of sites having both species prob cooccur probability that both species occur at a site exp cooccur expected number of sites having both species p lt probability that the two species would co-occur at a frequency less than the observed number of co-occurrence sites if the two species were distributed randomly (independently) of one another p gt probability of co-occurrence at a frequency greater than the observed frequency

### Abundance

**Figure 3:**
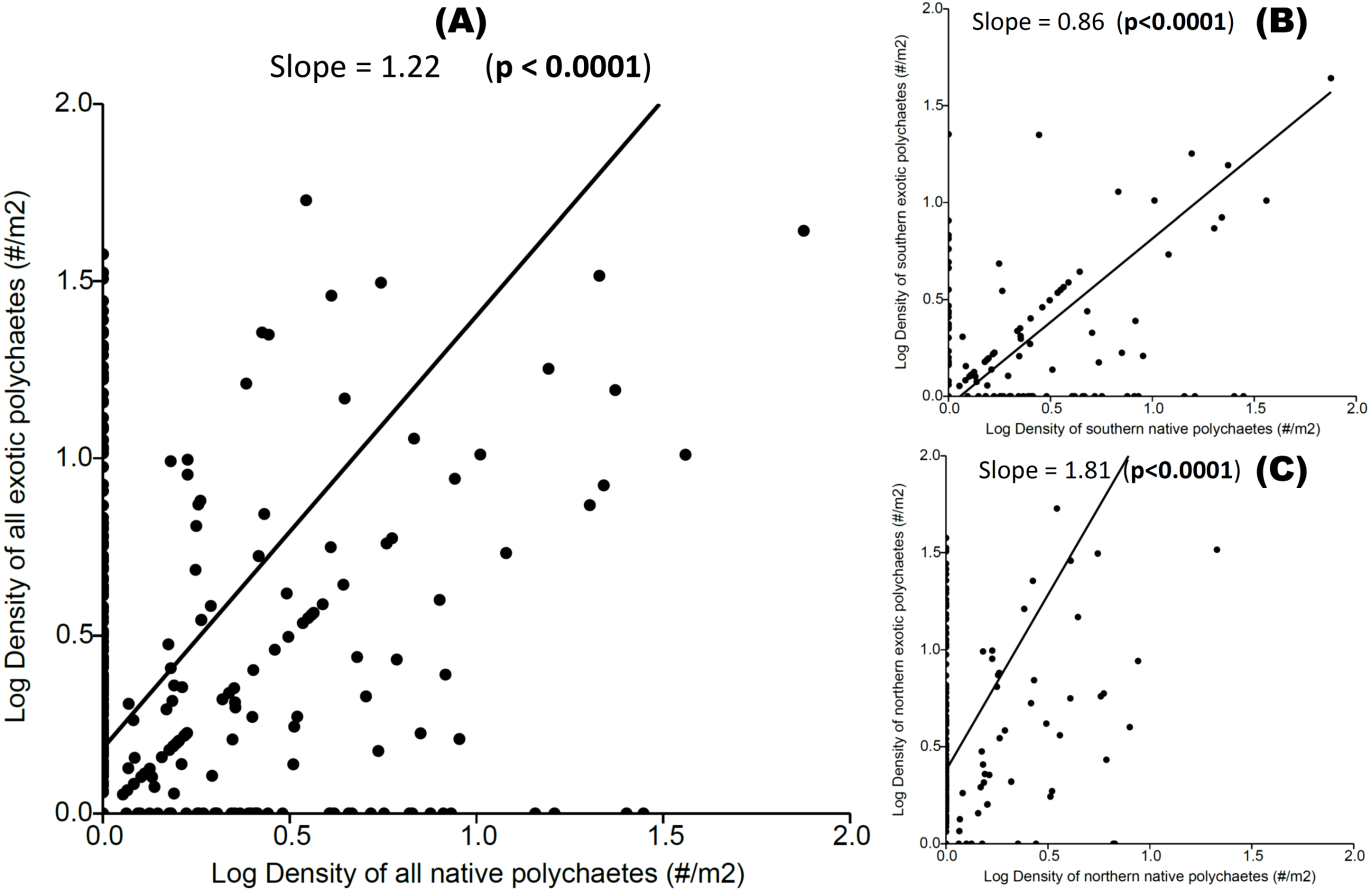
Relationship between native and exotic boring polychaete abundances (expressed as log transformed densities of polychaetes per host area). (A) All hosts and sites, (B) all hosts from the Southern area, and (C) all hosts from the Northern area. The slope and significance level are indicated on each graph.

We observed a significant positive relationship between exotic and native polychaete abundances at both local and regional scales (Fig. 3), indicating a coexistence trend. The transitive vs. intransitive competition analyses performed on *Transitivity* software support, at both local and regional scales, indicated the presence of a transitive network competition structure (i.e., All hosts and sites *τ*_p_ = 0.917, Mean = 0.884, limits = 0.583–1.000, Prop <1 = 0.410; All hosts from the Southern area *τ*_p_ = 0.667, Mean = 0.864, limits = 0.583–1.000, Prop < 1 = 0.452; and All hosts from Northern area *τ*_p_ = 0.750, Mean = 0.869, limits = 0.667–1.000, Prop < 1 = 0.308). In all of these cases the dominant eigenvector supports a transitive competition structure, where *P. rickettsi* is the dominant species at all sites (and in the northern region). At the regional scale *D. huelma* emerges as the dominant species in the southern region (Table 2).

## Discussion

Our results suggest that native and invasive boring polychaetes species coexist through weak competitive interactions (Hypothesis 2), together with transitive hierarchical competition (Table 2). This means that the boring polychate species present a hierarchical order in their competitive abilities, without evidence of positive species interactions (i.e., facilitation), allowing native and invasive species to coexist, as has been proposed by Bruno, Stachowicz & Bertness (2003). Our evidence of the importance of weak transitive competitive interactions allowing coexistence suggests that in many native communities where invasive species arrive, the expectation of competitive exclusion (Gause, 1934) may not be found because environmental resource availability prevents strong competition. From this perspective, weak competition, is a plausible alternative to competitive intransitivity networks for explaining species co-existence (Laird & Schamp, 2006; Soliveres et al., 2015; Ulrich, Jabot & Gotelli, 2017).

The exotic polychaete *P. rickettsi* interacts negatively with native and exotic species, suggesting a superior ability of this species to compete for resources. In this sense, life history characteristics can also be important for the outcome of some invasions (Kupferberg, 1997; Stohlgren et al., 1999), where invasive species not only have a superior ability to acquire resources but also more efficient resource conversion capacity (Byers, 2000). We propose that the competitive dominance of individuals of the exotic *P. rickettsi* is due to this species’ ability to monopolize space in host shells through the construction of galleries within the shell, which may impede the establishment of other individuals of boring polychaete species (e.g., exploitation competition). Indeed, *P. rickettsi* is a strict boring specialist that exclusively inhabits calcareous substrates and constructs U-shaped galleries, which may become more complex over time (Sato-Okoshi & Takatsuka, 2001; Diez, Radashevsky & Orensanz, 2011). Being a specialist in calcareous substrates, this species can monopolize the resource, resulting in the high levels of infestation found in different hosts (e.g., 52% Bertrán, Vargas & Quijón, 2005; 54% Diez, Radashevsky & Orensanz, 2011). This evidence, together with the observed positive relationship between native and exotic polychaete abundance, provides strong support for the hypothesis that *P. rickettsi* is a successful invader by being competitively dominant, but variations in local conditions prevent the exclusion of other boring polychaetes, potentially because this species is specialized and highly effective at boring on calcareous substrates, while the other species are more generalist.

**Table 2:** Observed and predicted (dominant eigenvector of the transition matrix) abundance distributions.

**Sampling**	**Speciestransitive competition structure**	**Observed relative Abundance**	**Dominant eigenvector**
**All hosts and sites**	*Polydora rickettsi*	0.561	1.000
	*Dipolydora huelma*	0.245	0.477
	*Boccardia tricuspa*	0.105	0.176
	*Dodecaceria opulens*	0.089	0.168
**Southern area**	*Dipolydora huelma*	0.474	1.000
	*Polydora rickettsi*	0.226	0.872
	*Boccardia tricuspa*	0.164	0.370
	*Dodecaceria opulens*	0.137	0.183
**Northern area**	*Polydora rickettsi*	0.800	1.000
	*Dipolydora huelma*	0.082	0.305
	*Boccardia tricuspa*	0.063	0.357
	*Dodecaceria opulens*	0.055	0.209

*Boccardia tricuspa* is the least specialized species in relation to the use of calcareous substrate (Moreno, Neill & Rozbaczylo, 2006), and it has been reported in different types of sandy, rocky and mud sediment substrates, as well as in coralline algae, between sponges and between tube-dwelling polychaetes (Rozbaczylo, Méndez & Bravo, 1994; Villamar, 2000; Villamar & Cruz, 2007). Therefore, by being more generalist in its use of resources, *B. tricuspa* is potentially less competitive on calcareous substrates, and it can survive in other substrates by niche differentiation, allowing coexistence by decreasing the competitive pression (i.e., weak competition hypothesis). Similarly, the interactions of *D. opulens* in this guild are not host specific, being associated with both multiple substrates, including shellfish valves and crustaceans (Rozbacyzlo & Carrasco, 1996; Hernández, Muñoz & Rozbaczylo, 2001; Moreno, Neill & Rozbaczylo, 2006), and also using calcareous substrates like a secondary borer (as reported for several species of the genus, (Martin & Britayev, 1998), or using cracks and crevices for refuge (Hernández, Muñoz & Rozbaczylo, 2001).

In this study even though the deterministic weak transitive competition is important for explaining the coexistence of native and exotic polychaetes, approximately 50% of the interactions observed in the boring polychaete guild were random. This means that species patterns can be accounted for by stochastic processes (Hubbell, 2001; Hubbell, 2006). This idea emphasizes that neutral-stochastic processes are important, and thus competition does not play a fundamental role, allowing species to accumulate randomly over time because: (1) there is a the high availability of resources (i.e., calcareous substrates for boring polychaetes); and (2) individuals from some species are randomly chosen from an assemblage with some probability of death (extinction), birth, migration and/or mutation. One consequence of this low-competition scenario is species coexistence, as observed in this study. Consequently, this guild of boring polychaetes could be functioning neutrally. The consideration of a broader conceptual framework that accommodates both biological structuring processes (niche based community assemblage by strong competitive interactions) and neutral stochastic processes (neutral based community assemblage with weak competitive interactions) in organizing species assemblages (Stokes & Archer, 2010; Alonso, Etienne & McKane, 2016) could provide a better understanding of the mechanisms limiting or enabling exotic species introductions.

Most other studies that evaluate the biotic resistance hypothesis do so by describing the relationship between native and exotic species diversity. However, this generalized approach can, and has, caused confusion (e.g., scale dependent results; Shea & Chesson, 2002; Byers & Noonburg, 2003) and has two potential drawbacks: (1) detection of the predicted negative pattern when the underlying mechanism is not species exclusion; and (2) not detecting the pattern when exclusion mechanisms are occurring that do not affect diversity, but do affect other ecological characteristics. In the first case, if a negative relationship in diversity is observed, one cannot immediately conclude negative biotic interactions, since this pattern could be the result of mechanisms such as habitat heterogeneity (“habitat checkerboards”), where species are associated with different abiotic features of the sites, leading to less co-occurrence than expected by chance (Gotelli & Entsminger, 2010). In this case, additional co-occurrence analyses, such as those used in this study, can provide a useful test to the competition hypothesis, where lower co-occurrence than expected by chance can be evidence of competitively structured communities. In the second case, the lack of a negative relationship, or weak negative relationship, between native and exotic diversity does not necessarily indicate that exclusion is not occurring; rather the mechanism may affect other variables, such as abundance. Especially in cases where total exclusion does not occur, as in this study, species abundance may be affected, which may not be detected by comparing native and exotic diversities. This situation can occur because competition rarely limits immigration or leads to local extinction (Levine, Adler & Yelenik, 2004; Bruno et al., 2005), therefore the result of negative interactions would only be observed in variables such as abundance, and may even result in coexistence, as seen in this study. Furthermore, in cases where the assemblage of interest is inherently species-poor (such as this study), the pattern may not be statistically detectable when diversity or richness is used as the response variable. In cases where no relationship is observed, either due to the lack of an effect on diversity or a lack of statistical power to detect this effect, analyses to evaluate other hypotheses (such as the biotic resistance hypothesis, positive species interactions, weak competitive interactions, or competitive intransitivity), can be supplemented by assessing the relationship between native and exotic abundances, and looking for constraints on abundance using transitivity analysis or other techniques (e.g., quantile regression).

## Conclusions

Our results support the idea that the response of a community to the arrival of exotic species is not necessarily given by changes in diversity (i.e., competitive exclusion; Levine, Adler & Yelenik, 2004), but rather by the relative importance of interactions (e.g., species interact negatively, such as *P. rickettsi* vs. all other species in this study, or randomly, such as all other species interactions in this study). This supports findings in the multispecies interactions with invaders in natural communities, where negative interactions are as common as positive ones (Henriksson et al., 2016). Coexistence may be a frequent result of interactions between native and exotic species, although perhaps less apparent than competitive exclusion. In this sense, the probabilistic point-of-view used in this study also provides a statistical tool for evaluating coexistence as a result of invasive and native species interactions, an idea which has been proposed in invasion ecology, but largely lacks empirical support and methodologies for detecting underlying mechanisms (Godoy, 2019).

Finally, we found support for the weak competition hypothesis, which is related to the weighted species richness hypothesis *sensu* Henriksson et al. (2016). This hypothesis describes biotic resistance as a weighted sum of the resident species, where the species-specific weights describe the relative contribution of each species to biotic resistance. In our study, we found evidence both in presence/absence and abundance data that *P. rickettsi* is a successful invader by being competitively dominant, but not excluding other species due to general weak competition. This evidence allows us to suggest that the low species richness of introduced species on the southeastern Pacific coast of Chile may be explained by the particular geographic isolation of this coast (i.e., low propagule pressure) and abiotic resistance (Castilla & Neill, 2009).

##  Supplemental Information

10.7717/peerj.8560/supp-1 Supplemental Information 1 Number of exotic and native boring polychaetes in native and exotic hosts from the southern Pacific coast of Chile

10.7717/peerj.8560/supp-2 Supplemental Information 2 Code to perform the Species Co-Occurrence Analysis in this study

10.7717/peerj.8560/supp-3 Supplemental Information 3 Code to perform the analysis in this study

## Additional Information and Declarations

### Field Study Permissions

The following information was supplied relating to field study approvals (i.e., approving body and any reference numbers): In Chile there is free access to all beaches, so no permits were needed for work on beaches. Whenever sampling occurred inside aquaculture facilities, we obtained the permission of the person in charge of the facility. The names of the managers/owners of the aquaculture facilities are: Oscar Alcalde Araya: Gerente de desarrollo y proyectos, Cultivos Marinos Caldera Ltda. Maximiliano Ramos: Cultivo San Sebastián Cristóbal Thompson/Sergio Uvillos: Cultivos Marinos San Cristobal S.A. Fabián Avilés, Fundación Chile

### Data Availability

The following information was supplied regarding data availability: The raw measurements are available in the Supplemental Files.
