# Supplementary material for: Patterns of association of native and exotic boring polychaetes on the southeastern Pacific coast of Chile: the combined importance of negative, positive and random interactions
Source: PeerJ. 2020 Apr 24;8:e8560. doi: 10.7717/peerj.8560 (PMC7203672; doi:10.7717/peerj.8560)
Supplement: Supplemental Information 3 [file peerj-08-8560-s003.docx]

**SUPPLEMENTARY MATERIAL**

**Supplementary material 2: R script**

**#** **COOCCUR script**

> Dat_TotalN1 <- read.csv("D:/Paula/Poliquetos/Total/Dat_TotalN1.csv", row.names=1, sep=";")

> View(Dat_TotalN1)

> library("cooccur", lib.loc="~/R/win-library/3.4")

> library("gridExtra")

> cooccur.Dat_TotalN1 <- cooccur(mat = Dat_TotalN1, type = "spp_site", thresh = TRUE, spp_names = TRUE)

# The cooccur() function produces an output object of class cooccur containing all of the results from the co-occurrence analysis

# Because the data are organized with species as rows and sites as columns (i.e., species by site) we can specify type = "spp_site" as a parameter to the function cooccur() and since we have species names we specify spp_names = TRUE

# According to their probabilities of co-occurrence some species in the dataset will be expected to share less than one site and it is recommended to filter these pairs from the analysis using thresh = TRUE

> class(cooccur.Dat_TotalN1)

> summary(cooccur.Dat_TotalN1)

# summary() method supplies a readout of the total positive, negative, and random species pairs classified by the algorithm. In addition, the function reports on the number of species and sites analyzed, the number of species pairs removed from the analysis by our threshold, and the number of species pairs that were not classifiable due to low statistical power

> prob.table(cooccur.Dat_TotalN1)

# method to obtain the complete set of species pairs analyzed and their probabilities

> plot(cooccur.Dat_TotalN1)

# This produces a visualization of all the pairwise combinations of species and their co-occurrence signs (positive or negative)

# The plot trims out any species that do not have any significant associations

> pair.attributes(cooccur.Dat_TotalN1)

# This function produces a table of the percentages of each species total pairings that were classified as positive, negative, or random

> pair.profile(cooccur.Dat_TotalN1)

# This function creates a box plot of the percentages of species pairs that were positive, negative, or random for all species

> cooccur(mat = Dat_TotalN1, type = "spp_site", thresh = FALSE, spp_names = TRUE, only_effects = TRUE, eff_standard = TRUE, eff_matrix = TRUE)

# Command to estimate the effect sizes or the differences between expected and observed frequencies of co-occurrence. These values can be standardized by dividing these differences by the number of sampling sites in the dataset.

# These values are bounded from -1 to 1, with positive values indicating positive associations and negative values indicating negative associations
